# Medical tourism in Thailand: a cross-sectional study

**DOI:** 10.2471/BLT.14.152165

**Published:** 2015-12-09

**Authors:** Thinakorn Noree, Johanna Hanefeld, Richard Smith

**Affiliations:** a International Health Policy Program, Ministry of Public Health, Tiwanon Road, Nonthaburi 11000, Thailand.; b Department of Global Health and Development, London School of Hygiene & Tropical Medicine, London, England.; c Faculty of Public Health and Policy, London School of Hygiene & Tropical Medicine, London, England.; Correspondence to Thinakorn Noree (email: thinakorn@ihpp.thaigov.net).

## Abstract

**Objective:**

To investigate the magnitude and characteristics of medical tourism in Thailand and the impact of such tourism on the Thai health system and economy.

**Methods:**

In 2010, we checked the records of all visits to five private hospitals that are estimated to cover 63% of all foreign patients. We reviewed hospital records of foreign patients and obtained data on their countries of origin, diagnoses and interventions. We surveyed 293 medical tourists to collect demographic characteristics and information on their expenditure and travelling companions. To help understand the impact of medical tourism on the Thai health system, we also interviewed 15 hospital executives and 28 service providers from the private hospitals.

**Findings:**

We obtained 911 913 records of hospital visits, of which 324 906 came from 104 830 medical tourists. We estimated that there were 167 000 medical tourists in Thailand in 2010. Of the medical tourists who attended our study hospitals, 67 987 (64.8%) came from the eastern Mediterranean region or Asia and 109 509 (34%) of them were treated for simple and uncomplicated conditions – i.e. general check-ups and medical consultations. The mean self-reported non-medical expenditure was 2750 United States dollars. According to the hospital staff interviewed, medical tourism in 2010 brought benefits to – and apparently had no negative impacts on – the Thai health system and economy.

**Conclusion:**

We estimate that the total number of medical tourists visiting Thailand is about 10% of previous national government estimates of 1.2 million. Such tourists appear to bring economic benefits to Thailand and to have negligible effects on the health system.

## Résumé

**Objectif:**

Étudier l'ampleur et les caractéristiques du tourisme médical en Thaïlande et l'impact de ce type de tourisme sur le système de santé et l'économie du pays.

**Méthodes:**

En 2010, nous avons examiné les dossiers de toutes les consultations réalisées dans cinq hôpitaux privés qui recevaient, selon les estimations, 63% de l'ensemble des patients étrangers. Nous avons consulté les dossiers d'hospitalisation des patients étrangers et recueilli des informations sur leur pays d'origine, le diagnostic et les interventions pratiquées. Nous avons interrogé 293 touristes médicaux afin d’en savoir plus sur leurs caractéristiques démographiques, leurs dépenses ainsi que leurs accompagnateurs. Afin de mieux comprendre l'impact du tourisme médical sur le système de santé thaïlandais, nous avons également interrogé 15 cadres hospitaliers et 28 prestataires de services de ces hôpitaux privés.

**Résultats:**

Nous avons examiné 911 913 dossiers de consultations en hôpital, dont 324 906 concernaient 104 830 touristes médicaux. Nous avons estimé que 167 000 touristes étaient venus en Thaïlande pour raison médicale en 2010. Parmi les touristes médicaux qui se sont rendus dans les hôpitaux de notre étude, 67 987 (64,8%) venaient de la région de la Méditerranée orientale ou d'Asie et 109 509 (34%) d'entre eux étaient traités pour des affections simples et sans complications – contrôles d'ordre général et consultations médicales. Les dépenses moyennes déclarées de nature autre que médicale s'élevaient à 2750 dollars des États-Unis. Selon le personnel hospitalier interrogé, en 2010, le tourisme médical a eu des effets bénéfiques - et apparemment pas d'impact négatif - sur le système de santé et l'économie thaïlandais.

**Conclusion:**

Nous estimons que le nombre total de touristes se rendant en Thaïlande pour raison médicale représente environ 10% des précédentes estimations nationales du gouvernement, qui étaient de 1,2 millions. Ce type de tourisme semble apporter des bénéfices économiques pour la Thaïlande, avec des effets négligeables sur le système de santé.

## Resumen

**Objetivo:**

Investigar la magnitud y las características del turismo sanitario en Tailandia y el impacto de dicho turismo sobre el sistema sanitario y la economía del país.

**Métodos:**

En 2010 se comprobaron los registros de todas las visitas a cinco hospitales privados que se estima que cubren el 63% de todos los pacientes extranjeros. Se revisaron los registros hospitalarios de los pacientes extranjeros y se obtuvieron datos de sus países de origen, diagnósticos e intervenciones. Se encuestó a 293 turistas sanitarios para recopilar características demográficas e información sobre sus gastos y compañeros de viaje. También se entrevistó a 15 ejecutivos de hospitales y 28 proveedores de servicios de hospitales privados para ayudar a entender el impacto del turismo sanitario en el sistema sanitario tailandés.

**Resultados:**

Se obtuvieron 911.913 registros de visitas hospitalarias, de los cuales 324.906 provenían de 104.830 turistas sanitarios. Se estimó que en 2010 hubo 167.000 turistas sanitarios en Tailandia. De los turistas sanitarios que atendieron los hospitales analizados, 67.987 (64,8%) provenían de la región del Mediterráneo Oriental o Asia y 109.509 (34%) fueron tratados de condiciones simples y sin complicaciones. Es decir, chequeos generales y consultas médicas. El promedio de gastos autodeclarados en gastos no médicos fue de 2.750 dólares estadounidenses. De acuerdo con el personal hospitalario entrevistado, el turismo sanitario en 2010 trajo beneficios a (y aparentemente no tuvo un efecto negativo sobre) el sistema sanitario y la economía de Tailandia.

**Conclusión:**

Se estima que el total de los turistas sanitarios que visitan Tailandia es cerca del 10% de las anteriores estimaciones del gobierno nacional de 1,2 millones. Esta clase de turistas parece traer beneficios económicos a Tailandia y no tienen efectos negativos en el sistema sanitario.

## ملخص

**الغرض:**

استقصاء حجم السياحة لأغراض طبية في تايلند وخصائصها وتأثير هذا النوع من السياحة على النظام الصحي والاقتصاد في البلاد.

**الطريقة:**

فحصنا سجلات جميع الزيارات في خمس مستشفيات خاصة تشمل خدماتها 63% من جميع المرضى الأجانب وفقًا للتقديرات، وذلك في عام 2010. وراجعنا سجلات المستشفيات المتعلقة بالمرضى الأجانب كما حصلنا على بيانات عن البلدان الأصلية التي ينتمون إليها، والتشخيصات، والتدخلات. واستطلعنا آراء 293 من السياح القادمين لأغراض طبية لجمع الخصائص الديموغرافية لهم والمعلومات المتعلقة بالنفقات ورفقاء السفر. كما أجرينا مقابلات مع 15 من المسؤولين التنفيذيين بالمستشفيات و28 من مقدمي الخدمات العاملين بالمستشفيات الخاصة، وذلك للمساعدة على استيعاب تأثير السياحة لأغراض طبية على النظام الصحي التايلندي.

**النتائج:**

حصلنا على 911,913 سجلاً لزيارات المستشفيات، ووجدنا أن 324,906 زيارة منها كانت من 104,830 وافدًا لأغراض السياحة. وتشير تقديراتنا إلى توافد 167,000 سائح لأغراض طبية في تايلند في عام 2010. وكان 67,987 (بنسبة 64.8%) من بين السائحين لأغراض طبية الذين قدموا إلى المستشفيات التي شملتها دراستنا قادمين من منطقة شرق البحر المتوسط أو آسيا، وتلقى 109,509 (بنسبة 34%) منهم العلاج فيما يتعلق بحالات بسيطة وغير معقدة – أي لمجرد إجراء فحوصات طبية عامة وبحثًا عن استشارات طبية. وبلغ متوسط النفقات غير الطبية وفقًا لما ذكره أصحابها 2,750 دولار أمريكي. وطبقًا لما ورد في المقابلات التي تم إجراؤها مع العاملين بالمستشفيات، عادت السياحة لأغراض طبية في عام 2010 بالنفع على النظام الصحي والاقتصاد التايلندي، دون أي تأثيرات سلبية بادية عليهما.

**الاستنتاج:**

وفقًا لتقديراتنا، بلغت نسبة إجمالي عدد السياح القادمين إلى تايلند لأغراض طبية حوالي 10% من 1.2 مليون وفقًا لتقديرات الحكومة الوطنية السابقة. وحقق هذا النوع من السياح بعض المنافع الاقتصادية لتايلند في ظاهر الأمر وكان له تأثيرات طفيفة على النظام الصحي.

## 摘要

**目的:**

旨在调查泰国医疗旅游的规模和特征，以及此类旅游对于泰国卫生系统和经济的影响。

**方法:**

在 2010 年，我们翻查了五家私立医院的所有就诊记录。据估计，这五家私立医院接诊的外国患者占所有外国患者总数的 63％。我们查看了外国患者的就医记录，并且获得了有关其原籍国、诊断和干预措施的资料。我们调查了 293 位医疗游客，收集了人口统计特征及其开支和旅伴方面的信息。为了帮助了解医疗旅游对泰国卫生系统的影响，我们还采访了私营医院中的 15 位医院主管和 28 位医务人员。

**结果:**

我们获得 911 913 项医院就诊记录，其中有 324 906 项来自 104 830 位医疗游客。我们估计 2010 年中有 167 000 位医疗游客入境泰国。在我们研究的医院中，接诊的 67 987 (64.8%) 位医疗游客来自东地中海地区和亚洲，其中 109 509 (34%) 位医疗游客的病情简单且不复杂，在治疗过程中接受了一般的体检和医疗咨询。平均自我报告的非医疗支出为 2750 美元。据接受采访的医院工作人员称，医疗旅游在 2010 年给泰国的卫生系统和经济带来了效益，显然没有任何负面影响。

**结论:**

我们估计，在国家政府先前估算的 120 万游客中，前往泰国的医疗游客总数约占 10％。此类游客显然给泰国带来了经济效益，对卫生系统产生的影响微不足道。

## Резюме

**Цель:**

Изучить масштабы и характеристики медицинского туризма в Таиланде и влияние туризма такого вида на систему здравоохранения и экономику Таиланда.

**Методы:**

В 2010 году были просмотрены записи всех посещений пяти частных больниц, которые, согласно подсчетам, обслуживают 63% всех иностранных пациентов. Были проанализированы больничные записи иностранных пациентов и получены данные о странах их происхождения, диагнозах и врачебном вмешательстве. Были опрошены 293 туриста-пациента, на основании чего получены демографические характеристики и сведения об их расходах и сопровождающих их лицах. Для оценки влияния медицинского туризма на систему здравоохранения Таиланда были также опрошены 15 руководителей больниц и 28 поставщиков услуг из частных больниц.

**Результаты:**

Были получены 911 913 записей о посещении больниц, 324 906 из которых поступили от 104 830 туристов-пациентов. Согласно подсчетам в 2010 году Таиланд посетили 167 000 туристов-пациентов. Из всех туристов-пациентов, посетивших исследуемые больницы, 67 987 (64,8%) прибыли из стран Восточного Средиземноморья или Азии, а 109 509 (34%) лечились от простых и неосложненных заболеваний, т. е. проходили общий медицинский осмотр и получали консультацию врача. Немедицинские расходы, о которых исследуемые пациенты сообщали самостоятельно, в среднем составили 2750 долларов США. По сообщениям опрошенных сотрудников больниц, медицинский туризм в 2010 году оказал положительное воздействие и, судя по всему, не имел негативного влияния на систему здравоохранения и экономику Таиланда.

**Вывод:**

Согласно нашим подсчетам общее количество туристов-пациентов, посетивших Таиланд, составляет приблизительно 10% от ранее определенного национальным правительством числа в 1,2 миллиона. Туристы, посещающие страну с такой целью, приносят экономическую пользу Таиланду и оказывают несущественное влияние на систему здравоохранения.

## Introduction

Although reliable estimates of the annual number of people travelling abroad to purchase medical services – so-called medical tourists – are difficult to identify, there has been a rise in medical tourism in the past decade.^1^ The increasing costs of health care and the expansion of the middle class in many low- and middle-income countries have led to an increase in such tourism.^2^^–^^4^

The global gross profit from medical tourism has been estimated to be about 60 billion United States dollars (US$) per year and to be growing by about 20% annually.^5^^,^^6^ Most studies of medical tourism have focused on the residents of North America, western Europe and the eastern Mediterranean region, many of whom have high purchasing power.^7^^,^^8^ In 2007, an estimated 50 000–120 000 residents of the United States of America travelled abroad to obtain medical services.^9^ In 2010, an estimated 63 000 residents of the United Kingdom of Great Britain and Northern Ireland also travelled abroad for medical care – mainly for fertility, cosmetic or bariatric treatments^10^ and predominately to Asia, eastern Europe, the Caribbean or South America.^11^^–^^14^

Medical tourism has often been portrayed as involving patients from high-income countries travelling to access cheaper care in low- and middle-income countries.^15^ However, a more complex market – with increasing numbers of medical tourists from low- and middle-income countries – is emerging.^16^^,^^17^ Regardless of the direction of travel, some middle-income countries have been positioning themselves as destination countries for medical tourism.^18^

The Thai Ministry of Commerce estimated that, in 2006, 1.2 million medical tourists accessed health services in Thailand and provided an estimated revenue of approximately US$ 1.1 billion – i.e. about 9% of Thailand’s total estimated revenue from tourism in 2006.^19^^,^^20^ Between the start of 2004 and end of 2008, medical tourism was estimated to have brought Thailand US$ 7.5 billion in revenue.^20^

Since 2003, the Thai Government has attempted to make Thailand a global centre for medical tourism through a Centre of Excellent Health Care of Asia initiative. Efforts at patient recruitment have included international road shows and tax exemptions for investment in new health facilities that target medical tourists.^21^

The Thai Government appreciates the potential of foreign patients as a source of foreign currency earnings. In 2011, it was estimated that revenues from medical tourists would generate the equivalent of 0.4% of Thailand’s gross domestic product.^19^ Despite such financial benefits, there is considerable concern about the equity impact of medical tourism, especially in areas where the health systems are weak and the resources may have to be diverted towards the care of patients from abroad.^19^ Another concern is the current lack of regulation and oversight of the providers of medical tourism.^22^ Ethical issues have been raised in relation to patients travelling after being given very limited information^23^ and foreign patients purchasing organs or surrogacy services from local populations in low- and middle-income countries.^24^^,^^25^ The world’s media recently described a boy who was born with Down syndrome to a surrogate mother in Thailand before being reportedly abandoned by his Australian parents.^26^

At present the division between those promoting the economic benefits of medical tourism and those convinced of the damaging effects of such tourism on health systems is unbridgeable because much of the discussion is based on poor empirical evidence.^27^^,^^28^ Here we attempt to provide further evidence by investigating the extent and the impact of medical tourism in Thailand.

## Methods

Five private hospitals were purposively selected for a survey. According to an unpublished survey of 55 hospitals conducted by the Thai Ministry of Commerce in 2007, the five selected hospitals together took 63% of all foreign patients visiting Thailand – with the remaining 37% diffusely distributed across the other 50 hospitals surveyed across the country (Department of International Trade Promotion, Thai Ministry of Commerce, unpublished observations, 2007). Three of the surveyed hospitals are in Bangkok and the other two are in the major tourist destinations of Chonburi and Phuket. Each surveyed hospital provides many foreign patients with highly specialized tertiary care and tailored service packages.

In a cross-sectional survey designed to identify all of the medical tourists who sought medical services at any of the surveyed hospitals in 2010, we analysed the records of all 911 913 hospital visits that occurred between 1 January 2010 and 31 December 2010. These records, which we retrieved from each hospital’s electronic database, included diagnoses coded according to the International Classification of Diseases, tenth revision (ICD-10).^29^ Foreign patients who had a permanent postal address in Thailand, had lived in Thailand for more than six months and/or were employed in Thailand were considered to be expatriates and not medical tourists. Following the advice of clinicians from the surveyed hospitals, foreign patients who had – according to their ICD-10 codes – presented with acute illnesses such as common cold and acute diarrhoea or as the result of minor accidents were assumed to be tourists who had fallen ill or been injured while on holiday in Thailand. All other foreign patients were considered to be medical tourists and their attendance records were analysed in terms of six demographic or service-use variables: (i) country of origin; (ii) treated as an inpatient or outpatient; (iii) diagnosis; (iv) type of procedure; (v) length of stay: and (vi) medical expenditure. To standardize data extraction among hospitals, we used ICD-9 clinical modification for the records that involved at least one procedure to code for the related organ system involved. 

Between May and August of 2012, to help understand the impact of medical tourism on the Thai health system, we conducted face-to-face semi-structured interviews with 43 key informants – i.e. 15 hospital executives and 28 service providers – from four of the surveyed hospitals that gave permission for such interviews. In the category of hospital executives, we interviewed chief executive officers, hospital directors, medical directors, human resources directors or marketing directors. The questions for this category assessed hospitals’ policies on serving foreign patients and on handling the revenue generated by these patients. In the category of service providers we interviewed doctors or nurses about how the services delivered to medical tourists differed from local residents.

In another series of interviews, we investigated medical tourists, mainly to assess their expenditure while in Thailand. We recruited these interviewees by consecutive case selection in the same hospitals as the key informants between June and September of 2012. We used a probability-proportional-to-size sampling technique to account for the between-hospital differences in the annual number of medical tourists. Our target – based on the available relevant data, a 5% level of precision and a 95% confidence interval – was to interview 578 medical tourists. Interviews were based on an unpublished questionnaire used by the Thai Ministry of Tourism and Sports for a survey of general tourist expenditure. This questionnaire was designed to collect demographic characteristics and information on the tourist’s expenditure and companions. The interviewers were nurses and translators from the hospitals, who had to take a half-day training course in data collection to standardize the interview process and minimize data collection errors. The interviews were held in Arabic, English or Japanese. All interviewees provided written informed consent.

The study protocol was approved by the ethics committees of the London School of Hygiene & Tropical Medicine and each study hospital. The initial findings were triangulated, for validity and the reduction of bias, in a presentation to policy-makers and related stake-holders in March 2014.

## Results

### Numbers of medical tourists

We identified 104 830 medical tourists who each visited one of the five surveyed hospitals in 2010. These tourists accounted for 44.3% of the 236 885 foreign patients identified in the hospital records and for 324 906 (35.6%) of the 911 913 attendances by foreigners. The mean number of visits per tourist was 3.1.

### Origins of medical tourists

The medical tourists were less likely to be residents of high-income countries in North America or Australasia than to be residents of the eastern Mediterranean or south-east or south Asia (Table 1). The highest numbers of medical tourists in Thailand in 2010 came from the United Arab Emirates (21 568), followed by Bangladesh (8443), the USA (7855) and Myanmar (7568); (Table 2).

**Table 1:** Areas of origin of the medical tourists attending five hospitals, Thailand, 2010

Origin	No. of medical tourists (%)
Eastern Mediterranean	40 554 (38.7)
South-east Asia	14 730 (14.1)
Europe	14 004 (13.4)
South Asia	12 703 (12.1)
North America	9 481 (9.0)
East Asia	4 166 (4.0)
Africa	3 957 (3.8)
Australasia	3 949 (3.8)
Unknown	1 252 (1.2)
Other area	34 (0.0)
**Total**	**104 830 (100.0)**

**Table 2:** Origins of medical tourists attending five hospitals, Thailand, 2010

Rank	Origin	No. (%)		Mean no. of visits per medical tourist
		Medical tourists (*n* = 104 830)	Visits (*n* = 324 906)	
**1**	United Arab Emirates	21 568 (20.57)	63 457 (19.53)	2.94
**2**	Bangladesh	8443 (8.05)	26 338 (8.11)	3.12
**3**	United States	7855 (7.49)	24 262 (7.47)	3.09
**4**	Myanmar	7568 (7.22)	32 940 (10.14)	4.35
**5**	Oman	7096 (6.77)	21 699 (6.68)	3.06
**6**	Qatar	5212 (4.97)	17 784 (5.47)	3.41
**7**	United Kingdom	3935 (3.75)	10 779 (3.32)	2.74
**8**	African countries other than South Africa^a^	3857 (3.68)	17 491 (5.38)	4.53
**9**	Cambodia	3837 (3.66)	10 919 (3.36)	2.85
**10**	Australia	3360 (3.21)	10 136 (3.12)	3.02
**11**	Kuwait	3159 (3.01)	11 330 (3.49)	3.59
**12**	Japan	1995 (1.90)	4681 (1.44)	2.35
**13**	France	1742 (1.66)	4275 (1.32)	2.45
**14**	Germany	1545 (1.47)	3780 (1.16)	2.45
**15**	Canada	1474 (1.41)	4115 (1.27)	2.79

^a^ The hospitals categorized the origins of African patients as “South Africa” or “other African countries”.

Note: The table only represents the top 15 countries.

### Procedures

**Fig. 1:**
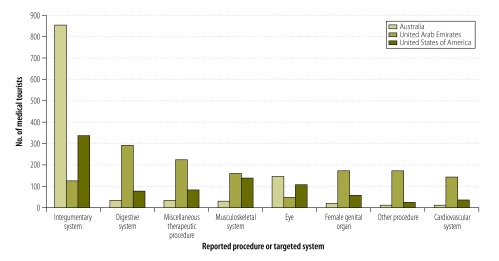
Most commonly-recorded procedures or targeted systems among medical tourists from three countries attending five hospitals, Thailand, 2010

Notes: The procedures targeted at the integumentary system were mostly cosmetic. Cardiac catheterizations accounted for most of the “miscellaneous therapeutic procedures” and angiocardiograms accounted for most of the “other procedures”.

Fig. 1 shows the organ system involved in medical or surgical services purchased by medical tourists from Australia, the United Arab Emirates and the USA. Medical tourists from the USA most frequently purchased cosmetic surgery procedures (on the integumentary system), followed by orthopaedic operations (musculoskeletal) and eye operations. Cosmetic surgery accounted for most of the services purchased by Australian patients, while patients from the United Arab Emirates primarily purchased digestive system surgeries.

### Impacts

**Fig. 2:**
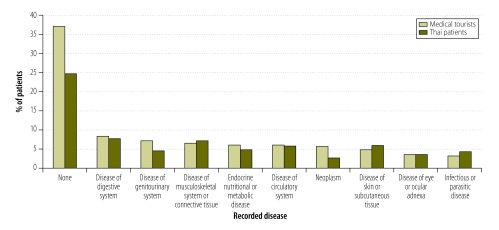
Most commonly-recorded diseases of medical tourists and Thai patients attending five hospitals, Thailand, 2010

Note: When no disease was reported, the tourist or Thai patient was usually attending hospital solely for a health check-up or medical consultation.

The 104 830 medical tourists represented approximately 14% of the 734 150 patients seen at our five surveyed hospitals in 2010. They represented a small proportion of the private patients seen in hospitals across Thailand in the same period – and an even smaller proportion of the patients seen at any Thai hospital. Furthermore, of the 324 906 visits by medical tourists, 303 167 (93%) were outpatient-only and 109 509 (34%) were only for a general check-up, a medical consultation or for the treatment of a simple, uncomplicated condition (Fig. 2).

Interviews with hospital executives and service providers indicated no difference in the key aspects of clinical care between foreign and domestic patients. Whatever their origins, patients were reportedly treated according to the same medical guidelines. However, there were some differences in terms of peripheral services, such as provision of a translator, transfer services from the airport and special food for medical tourists. Difficulties in communication often meant that doctors and nurses spent more time with a medical tourist than with a domestic patient. However, all of our interviewees were adamant that the reported differences – and medical tourism in general – did not affect the overall quality of medical services that they provided to any patient.

### Non-medical expenditure

Time constraints of the study meant that we only interviewed 293 medical tourists – i.e. 50.7% of the target sample. Only 100 (34%) had arranged to visit Thailand solely for medical services. Another 54 (18%) had decided to seek medical care in the country after they had arrived. About half (151) had travelled to Thailand with at least one companion.

The medical tourists we interviewed reported a mean non-medical expenditure for themselves of US$ 2750, and US$ 2678 for their companions – spent mainly on accommodation, food, drinks and shopping. Medical tourists and companions who had travelled on long-haul flights – e.g. from Australasia, Europe and North America – spent an average of US$ 2142 and US$ 2387, respectively, on traditional tourist activities within Thailand. This was considerably less than the corresponding values – of US$ 3031 and US$ 2800, respectively – for the medical tourists and companions who had travelled shorter distances within Asia.

Using our estimated numbers and expenditures, we estimated that the gross tourism revenue from medical tourists and their companions in 2012 was approximately US$ 900 million.

## Discussion

Together the five surveyed hospitals had been estimated to handle 63% of medical tourists in Thailand, which would indicate from our results that there were about 167 000 medical tourists who attended Thai hospitals in 2010. Our estimate of medical tourists falls far below the national government estimate of 1.2 million in 2006^19^ and puts into doubt the accuracy of the projected figure of some 7 million foreign patients being seen in Thailand in 2015.^21^ Earlier estimates have probably been affected by the failure to separate medical tourists from expatriates, immigrants and tourists who have simply fallen ill or been injured while on holiday in Thailand. Another potential cause of inaccuracy is that Thai hospitals traditionally report the number of foreign patients by the number of visits – rather than the number of patients – and, as we observed, each foreign patient may account for multiple visits in any year. The possibility remains that other national and global estimates of the numbers of medical tourists are also far too high.

It was once thought that most medical tourists from North America and Europe were seeking complicated procedures when they travelled to Thailand for medical care.^30^ However, this study and others have found that most such tourists travel to Thailand for minor elective procedures, such as cosmetic surgery.^31^ In contrast, we found that medical tourists visiting Thailand from the United Arab Emirates were generally purchasing services such as cardiac catheterization, angiocardiograms, other cardiovascular procedures and gastric bypasses.

The Thai health system is predominantly public, with a relatively small proportion of patients visiting private health facilities.^32^ In 2008, public hospitals accounted for 80% of beds,^33^ although private hospitals have a proportionately larger role in Bangkok and other urban centres. Of the 35 789 physicians working in Thailand in 2009, 82.9% worked in the public sector.^33^ Although Thailand has 18 public medical schools and one private one – that together produce about 2500 new graduates annually,^34^ there remains a shortfall in doctors. It has been estimated that at the current rate of training, Thailand will not achieve its targeted physician density – of one per 1500 people – until 2020.^34^ The situation is compounded by the distribution of doctors, with an acute shortage in rural areas. In 2009, there was one doctor per 565 people in Bangkok but only one per 2870 people in the north-eastern provinces.^33^ This inequitable distribution may be made worse by doctors moving from tertiary public hospitals to urban private hospitals. Part of this flow has been attributed to the large numbers of medical tourists attending urban private hospitals.^21^^,^^32^

There have been few empirical studies examining the impacts of medical tourism in Thailand on the health system. In 2006, it was estimated that 176–303 additional doctors would be required to service foreign patients by 2015 – i.e. 9–12% of the additional doctors that were estimated to be needed for the entire national health system.^21^ In 2011, however, it was estimated that far more additional doctors – i.e. 528–909 – would be required to service foreign patients by 2015.^19^ However, both of these estimates of the numbers of additional doctors required were based on estimates of the numbers of medical tourists that now appear far too high. Our survey and interview results indicate that medical tourism is on such a small scale in Thailand that its effect on the domestic health system is marginal. If such tourism does have any detrimental effect it is likely to be primarily via its impact on the distribution of doctors. Most of the doctors and nurses who work in private hospitals in Thailand have been trained in the public sector, through the use of public funds. However, the results from this study indicate that it is the provision of private health care to residents of Thailand that drives most of the flow of doctors to the private sector.

Medical tourists make a net contribution to the domestic economy, not only in terms of their medical spending but also, frequently, in terms of their spending on traditional tourist activities. In 2012, the Ministry of Tourism and Sports estimated that each non-medical tourist spent a mean of US$ 803^35^ whereas – according to our interviews and excluding any medical expenditure – the medical tourists and their companions each spent more than three times this figure in the same year. It is unclear why the distance travelled to Thailand should affect non-medical expenditure in Thailand but one possibility is that the long-haul medical tourists and companions are, in general, less affluent compared with their short-haul counterparts and so spend less on accommodation, food, drinks and shopping.

## Conclusion

Medical tourists in Thailand do not form a homogeneous group. They are a mix of patients who travel with serious health issues and those seeking minor treatments while taking a holiday. Their numbers have been over-estimated in the past. They appear to have a negligible effect on the national health system but do contribute to the Thai tourism industry. A medical tourist tax, to channel some of the proceeds from the tourism industry into the health system, could be considered to redistribute some of these gains to the sector that is providing the relevant services.

## References

[1] Connell J. Contemporary medical tourism: conceptualisation, culture and commodification. Tourism Manage. 2013;34:1–13. 10.1016/j.tourman.2012.05.009

[2] Bies W, Zacharia L. Medical tourism: outsourcing surgery. Math Comput Model. 2007;46(7-8):1144–59. 10.1016/j.mcm.2007.03.027

[3] Hopkins L, Labonté R, Runnels V, Packer C. Medical tourism today: what is the state of existing knowledge? J Public Health Policy. 2010 7;31(2):185–98. 10.1057/jphp.2010.10 20535101

[4] Horowitz MD, Rosensweig JA. Medical tourism – health care in the global economy. Physician Exec. 2007 Nov-Dec;33(6):24–6,. 18092615

[5] Heung V, Kucukusta D, Song H. Medical tourism development in Hong Kong: an assessment of the barriers. Tourism Manage. 2011;32(5):995–1005. 10.1016/j.tourman.2010.08.012

[6] Herrick MD. Medical tourism: global competition in healthcare. Dallas: National Center for Policy Analysis; 2007. Available from: http://www.ncpa.org/pdfs/st304.pdf [cited 2015 Sep 13].

[7] Medical tourism: consumers in search of value. Washington: Deloitte Center for Health Solutions; 2008. Available from http://www.academia.edu/9144718/Medical_Tourism_Consumers_in_Search_of_Value_Produced_by_the_Deloitte_Center_for_Health_Solutions [cited 2015 Sep 10].

[8] Connell J. Medical tourism: sea, sun, sand and surgery. Tourism Manage. 2006;27(6):1093–100. 10.1016/j.tourman.2005.11.005

[9] Johnson TJ, Garman AN. Impact of medical travel on imports and exports of medical services. Health Policy. 2010 12;98(2-3):171–7. 10.1016/j.healthpol.2010.06.006 20619919

[10] Hanefeld J, Horsfall D, Lunt N, Smith R. Medical tourism: a cost or benefit to the NHS? PLoS ONE. 2013;8(10):e70406. 10.1371/journal.pone.0070406 24204556 PMC3812100

[11] Tattara G. Medical tourism and domestic population health [working paper]. Venice: University of Venice; 2010. Available from: http://papers.ssrn.com/sol3/papers.cfm?abstract_id=1544224 [cited 2014 Aug 12].

[12] Connell J, Fara X. Medical tourism in the Caribbean Islands: a cure for economies in crisis? Isl Stud J. 2013;8:115–30.

[13] Lautier M. Export of health services from developing countries: the case of Tunisia. Soc Sci Med. 2008 7;67(1):101–10. 10.1016/j.socscimed.2008.01.057 18468756

[14] Kangas B. Hope from abroad in the international medical travel of Yemeni patients. Anthropol Med. 2007;14(3):293–305. 10.1080/13648470701612646 27268744

[15] Mazzaschi A. Surgeon and safari: producing valuable bodies in Johannesburg. Signs (Chic). 2011;36(2):303–12. 10.1086/655941 21114075

[16] Crush J, Chikanda A. South-South medical tourism and the quest for health in southern Africa. Soc Sci Med. 2015 1;124:313–20. 10.1016/j.socscimed.2014.06.025 24973022

[17] Lunt N, Horsfall D, Smith R, Exworthy M, Hanefeld J, Mannion R. Market size, market share and market strategy: three myths of medical tourism. Policy Polit. 2014;42(4):597–614. 10.1332/030557312X655918

[18] Leng CH. Medical tourism and the state in Malaysia and Singapore. Glob Soc Policy. 2010;10(3):336–57. 10.1177/1468018110379978

[19] NaRanong A, NaRanong V. The effects of medical tourism: Thailand’s experience. Bull World Health Organ. 2011 5 1;89(5):336–44. 10.2471/BLT.09.072249 21556301 PMC3089382

[20] Thailand: medical hub of Asia [Technical report]. Nonthaburi: Office of the Civil Servant Commission; 2010.

[21] Pachanee CA, Wibulpolprasert S. Incoherent policies on universal coverage of health insurance and promotion of international trade in health services in Thailand. Health Policy Plan. 2006 7;21(4):310–8. 10.1093/heapol/czl017 16728511

[22] Smith R, Lunt N, Hanefeld J. The implications of PIP are more than just cosmetic. Lancet. 2012 3 31;379(9822):1180–1. 10.1016/S0140-6736(12)60166-4 22305764

[23] Snyder J, Crooks VA. Medical tourism and bariatric surgery: more moral challenges. Am J Bioeth. 2010 12;10(12):28–30. 10.1080/15265161.2010.528510 21161839

[24] Crush J, Chikanda A, Maswikwa B. Patients without borders: medical tourism and medical migration in southern Africa. Cape Town: Megadigital; 2012.

[25] Whittaker A, Speier A. “Cycling overseas”: care, commodification, and stratification in cross-border reproductive travel. Med Anthropol. 2010 10;29(4):363–83. 10.1080/01459740.2010.501313 21082483

[26] Baby Gammy may be given Australian citizenship, government suggests. The Guardian. 2014 Aug 4. Available from: http://www.theguardian.com/world/2014/aug/04/baby-gammy-australian-citizenship-government-suggests [cited 2014 Aug 5].

[27] Hanefeld J, Smith R, Horsfall D, Lunt N. What do we know about medical tourism? A review of the literature with discussion of its implications for the UK National Health Service as an example of a public health care system. J Travel Med. 2014 Nov-Dec;21(6):410–7. 10.1111/jtm.12147 25156070

[28] Smith R, Martínez Álvarez M, Chanda R. Medical tourism: a review of the literature and analysis of a role for bi-lateral trade. Health Policy. 2011 12;103(2-3):276–82. 10.1016/j.healthpol.2011.06.009 21767892

[29] International statistical classifications of diseases and related health problems, 10th revision. Geneva: World Health Organization; 2010. Available from: http://apps.who.int/classifications/icd10/browse/2010/en [cited 2014 Jul 18].

[30] Executive summary: a development of Thailand medical hub policy. Nonthaburi: Ministry of Public Health; 2010.

[31] Noree T, Hanefeld J, Smith R. UK medical tourists in Thailand: they are not who you think they are. Global Health. 2014;10(1):29. 10.1186/1744-8603-10-29 24885204 PMC4038702

[32] Wibulpolprasert S, Pengpaibon P. Integrated strategies to tackle the inequitable distribution of doctors in Thailand: four decades of experience. Hum Resour Health. 2003 11 25;1(1):12. 10.1186/1478-4491-1-12 14641940 PMC317381

[33] Thailand health profile 2008–2010. Nonthaburi: The War Veterans Organization of Thailand; 2012.

[34] Suphanchaimat R, Wisaijohn T, Thammathacharee N, Tangcharoensathien V. Projecting Thailand physician supplies between 2012 and 2030: application of cohort approaches. Hum Resour Health. 2013;11(1):3. 10.1186/1478-4491-11-3 23374250 PMC3575403

[35] Report on international tourists in Thailand in 2012. Nonthaburi: Ministry of Tourism and Sports; 2012. Available from: http://www.tourism.go.th/home/details/11/222/2137 [cited 2015 Sep 13].

